# Eating rate has sustained effects on energy intake from ultraprocessed diets: a 2-week ad libitum dietary randomized controlled crossover trial

**DOI:** 10.1016/j.ajcnut.2025.11.012

**Published:** 2025-11-26

**Authors:** Ciarán G Forde, Lise AJ Heuven, Marieke van Bruinessen, Zhen Liu, Markus Stieger, Kees de Graaf, Marlou P Lasschuijt

**Affiliations:** Sensory Science and Eating Behavior Group, Division of Human Nutrition and Health, Wageningen University & Research, Wageningen, The Netherlands

**Keywords:** dietary energy intake, food texture, eating rate, ultraprocessed diet, nova, metabolic health

## Abstract

**Background:**

Previous research has shown that diets dominated by ultraprocessed foods (UPF) are associated with higher dietary energy intakes. This association may be attributable in part to meal texture and associated eating rate (ER). Experimental studies are needed to clarify the underlying mechanisms.

**Objectives:**

The objective was to compare daily energy intake (kcal/d) from diets with different meal texture-derived ER (g/min) (UPF Slow-ER compared to UPF Fast-ER) across a 14-day period. Two UPF diets comprised of foods selected to have textures known to lead to a slower or faster ER.

**Methods:**

Forty-one participants [*n =* 21 male, mean (± standard deviation) age 27 ± 5; weight 70 ± 10 kg; body mass index 23.4 ± 1.9] completed a single-blind, block-randomized crossover study including 2 14-day diets; UPF Slow-ER and UPF Fast-ER, with a 2-wk washout. Diets were served ad libitum and matched for palatability, portion size served, total energy served, non-beverage energy density, and meal variety.

**Results:**

Daily energy intake was 369 kcal/d (95% confidence interval: 221, 517) lower on the UPF Slow-ER diet compared with the UPF Fast-ER diet [main effect; F (1, 1051) = 23.98, *P* < 0.001]. The effect on energy intake was consistent across participants and the number of days on the diet [diet∗time: F (13, 1051) = 0.96, *P* = 0.486], and was not attributable to meal liking or macronutrient intake (all, *P* > 0.05). There was no change in body weight pre-diets to post-diets, and no differences in body weight between the 2 diets. Body fat mass decreased on the UPF Slow-ER diet by 0.43 kg [main effect; F (1, 119) = 14.68, *P* = 0.0002].

**Conclusions:**

Food texture-derived ER has a significant and sustained effect on energy intake of ultraprocessed diets over a 2-wk period. This finding highlights the importance of food texture in guiding ER and the central role of sensory cues in regulating meal size.

This trial was registered at clinicaltrials.gov as NCT06113146.

## Introduction

In recent years, there has been an increased interest in the specific aspects of the food supply that contribute to positive energy balance, beyond simply the nutrient composition of diets alone. One aspect that has received a lot of attention is the degree to which a food has been processed, as this has been reported in numerous dietary observational studies to be associated with weight status [1]. Ultraprocessed foods (UPFs) have been defined by the Nova classification scheme as “industrial formulations that are made entirely from food derivatives, chemical substances and sequence of processes, that bears little resemblance to the original food material” [2]. The long-term effects of diet quality on health have traditionally been assessed with a focus on the nutrient quality and composition of foods that inform our dietary patterns [3,4]. A food’s sensory cues are rarely considered in terms of their effect on health, whereas these cues are known to drive meal-to-meal variations in food selection, amount of food consumed and can inform our habitual dietary patterns [5]. Food odors can drive sensory-specific appetites, taste promotes acceptance, and signals the predominant nutrients being consumed, and food texture has been shown to influence meal size through eating rate (ER, g/min) [[5], [6], [7], [8], [9]]. Food texture informs the oral processing behaviors needed to form a ready-to-swallow food bolus, and drives microstructural patterns of consumption such as bite size, chews/bite and oro-sensory exposure time (in-mouth duration of the food bolus) that ultimately inform our ER (g/min). A slower ER has been shown to influence downstream physiological responses to the food consumed and is related to food digestion, metabolism of nutrients consumed and satiation [5,[10], [11], [12]]. When food is ingested quickly (Fast-ER), this leads to higher food intakes, lower satiety and if sustained this leads to caloric overconsumption and positive energy balance [[13], [14], [15]].

Findings from dietary epidemiological studies and the results of a single randomized controlled diet trial (RCT) have demonstrated a positive association between higher UPF consumption and greater energy intakes [1,16]. The RCT compared energy intake across 2 wk on a minimally processed or UPF diet and reported an average net difference of 508 kcal/d greater energy intake between the 2 diets [16]. One of the proposed mechanisms by which UPFs may have promoted higher intakes is through faster ERs [17]. The UPF diet of that trial had a faster ER and a higher non-beverage energy density that combined led to large differences in energy intake rate (kcal/min) between the UPF and minimally processed diet [16]. Results from 2 follow-up studies further support this hypothesis; that texture-based faster ERs promote higher food and energy intakes for meals and diets, independently of the foods’ degree of processing (UPF or minimally processed) [18,19]. Results from these acute studies demonstrate the impact of texture-derived differences in ER on food and energy intake within a single day and could potentially explain the findings of the UPF RCT that showed diet-level differences in energy intake between the tested UPF and minimally processed diets that differed in ER.

We conducted a block-randomized controlled diet intervention trial to compare the sustained impact of meal ER on energy intakes from UPF diets. The diets were based on a selection of nutritionally similar UPF foods with differing textures, known to produce differences in ER. The primary objective was to determine the effect of meal texture-derived differences in ER (g/min) of ultraprocessed diets (UPF Slow-ER compared to UPF Fast-ER) on daily ad libitum energy intake (kcal/d) across a 14-day period. We hypothesized that the energy intake from UPF diets is affected by meal texture-derived differences in ER, such that when meals are consumed at a slower ER, they will lead to lower daily energy intake compared with a nutritionally comparable UPF diet consumed with a faster ER. The secondary objectives were to compare appetite and body composition changes when on a 14-day diet of UPF with either a slow or a Fast-ER. Our hypothesis for these secondary outcomes is that a diet of UPF with a Slow-ER will lead to sustained reductions in energy intake without a notable difference in reported appetite and that this would support a small reduction in body weight and adiposity compared with the UPF diet with a faster ER.

## Methods

### Study design

**FIGURE 1:**
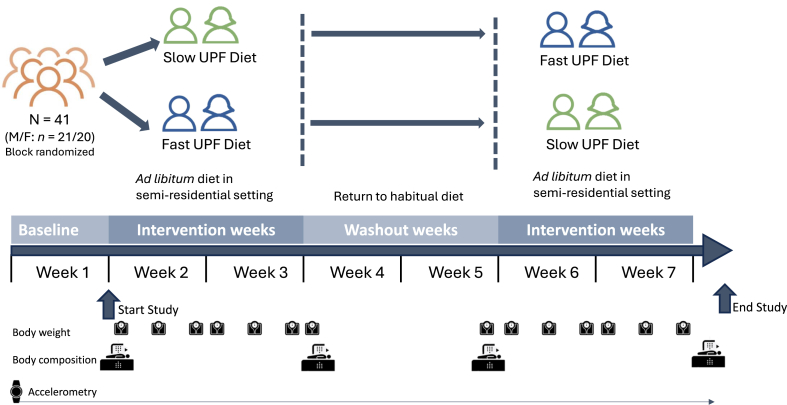
Overview of the restructure study design. ER, eating rate; UPF, ultraprocessed food.

The Restructure RCT was a single-blind, block-randomized crossover diet intervention trial with 2 14-day diet arms separated by an equivalent washout period. In a semiresidential setting, participants were offered 2 diets comprising UPFs (∼95% energy from UPF) which consisted of 14 d of measured ad libitum intake on a diet with a Slow-ER (UPF Slow-ER) or a 14-day ultraprocessed diet with a Fast-ER (UPF Fast-ER). All participants received both diets in randomized order (crossover, within-subject design). The study included a 1-wk run-in period during which habitual dietary intake and activity levels were assessed. This was followed by a 14-day period on one of the intervention diets, a washout period of 14 d to reduce potential carry-over effects, and finally the second 14-day diet period on the other intervention diet (Figure 1).

During the 2 intervention diet periods, participants were provided all foods in ad libitum portions, and energy intake at every meal and snack was measured through preconsumption, and postconsumption weighing to determine food consumed (g and kcal). Body weight and composition were measured at baseline, after each intervention period and at the end of the washout period (Figure 1). A detailed description of the study methods and rationale is presented in the study protocol [20]. The study was conducted between January and November 2024 at the Human Nutrition Research Unit of Wageningen University and Research, The Netherlands. The study was approved by the Medical Ethical Committee “East-Netherlands,” The Netherlands (ABR: NL83462.091.23) and complied with the Declaration of Helsinki for Medical Research involving Human Subjects. The study was preregistered at clinicaltrials.gov (NCT06113146), and the data analysis plan was preregistered at Open Science Framework (https://osf.io/afmkh).

### Participants

Participants were recruited via social media, flyers, and mailing lists. Participants were eligible to participate if they were aged between 21 and 50 y, had a BMI (in kg/m^2^) between 21 and 27, reported good general and mental health, reported normal appetite, and consumed 3 main meals a day at regular times. Participants were excluded if they were allergic, intolerant, or not willing to consume any of the test foods, did not like >20% of the foods included in the diet (screened on recruitment), were smokers, used medication that include hormone therapy or affect the immune system or any medication that affected study outcomes; food intake, appetite or metabolic responses as assessed by a research physician, had excessive alcohol consumption (≥21 glasses/wk for males and ≥14 glasses/wk for females), were not weight stable in the past half year, did >4 h/wk moderate-to-vigorous physical activity, or if their habitual food intake of UPF was higher than 50% of daily food intakes as measured with a newly developed UPF food frequency questionnaire (van Bruinessen et al. 2025 under review).

Prospective participants were invited to an information session, and after signing informed consent, participants filled out a general health questionnaire to screen for the inclusion and exclusion criteria [20]. Body weight (SECA 635 platform scale) and height (SECA stadiometer) were measured in duplicate to assess BMI. Potentially eligible participants were invited for a screening session to measure fasted glucose level (inclusion range: 3.5–8 mmol/L), Hb value (inclusion range; 7.5–11.0 mmol/L female and 8.5–11.0 mmol/L male) blood pressure (excluded if below 90 and/or below 60 mm hg) and natural ER using the carrot test (inclusion range, 5–35 g/min) [21]. In addition, participants tasted a series of samples of the foods included in the 2 diets and rated their liking and familiarity. Participants were excluded if they did not like > 20% of the foods (rated ≤4 on a 9 point-hedonic scale, based on tasting).

### Study diets

The diets consisted of commercially available, familiar, Dutch foods that would be classified as ultraprocessed based on the Nova scheme (Table 1) [2]. The UPF fast and UPF slow ad libitum diets were developed based on ER data from previous research [[17], [18], [19],[22], [23], [24], [25], [26], [27]] (van Bruinessen et al. 2025 under review). The degree of processing was defined using the Nova classification [2] and Nova-class for each meal and ingredient was assigned by 2 independent coders. There was disagreement for 7% of the breakfast foods, 2% of the lunch and 9% of the dinner foods. These ingredients were classified by a third coder to reach consensus.

**TABLE 1:** Diet composition of the average day menu presented to the participants during the UPF Slow-ER and UPF Fast-ER diet periods

	UPF Slow-ER	UPF Fast-ER	Average of both diets
Average daily meals excl. snacks			
Amount (g)	3324	3326	3325
Total energy (kcal)	5062	5066	5064
Energy density (kcal/g)	1.52	1.52	1.52
Fat (EN%)	22	34	28
Carbohydrates (EN%)	52	45	49
Monodisaccharides and disaccharides (EN%)	13	17	15
Protein (EN%)	22	18	20
Fiber (g/100 kcal)	1.54	1.52	1.53
Sodium (Na) (mg/100 kcal)	168	176	172
Water content^1^ (g/100 g)	65	67	66
Ultraprocessed foods^2^ (EN%)	98	94	96
Average daily meals incl. snacks^3^			
Amount (g)	3821	3823	3822
Energy (kcal)	5831	5835	5833
Energy density (kcal/g)	1.53	1.53	1.53
Fat (EN%)	22	33	28
Carbohydrates (EN%)	53	47	50
Mono and disaccharides (EN%)	15	19	17
Protein (EN%)	21	16	18
Fiber (g/100 kcal)	1.54	1.48	1.51
Sodium (mg/100 kcal)	408	407	407
Water content^1^ (g/100 g)	65	67	66
Ultraprocessed foods^2^ (EN%)	97	94	95

Abbreviations*:* EN, energy; ER, eating rate; UPF, ultraprocessed food.

1 Estimated by subtracting the fat, carbohydrates, fiber, protein, and salt content in grams from the total amount in grams.

2 Ultraprocessed foods, defined using the Nova classification; calculated as the weighted average of the Nova classification of the ingredients.

3 Including fruits, snacks, and weekend soda drinks.

Each diet comprised 7 unique daily menus (breakfast, lunch, and dinner) which were presented twice within each diet period. The 2 diets were nutritionally matched such that in both diets meals were the same in terms of portion size served (gram), variety (number of meal components), total energy served (kcal), meal energy density (kcal/gram), visual volume, liking and familiarity [28]. Energy and nutrient intake were calculated based on packaging information, and if unavailable the Dutch food composition table (Dutch: Nederlands Voedingsstoffenbestand (NEVO) table 2019, version 6) was used. The average energy density of the diets was 1.53 kcal/g, similar to the average Dutch diet (1.55 kcal/g) [29]. The diets were matched for fiber, sodium, and macronutrient content on the level of the 2-wk diet (not on meal- or day menu level). We gave priority to matching diets on variables matched on meal level, with specific priority to matching nonbeverage energy density, because this can exert a dominant effect on intake. This resulted in some differences in the macronutrient matching of the UPF slow compared with the fast diet when compared in the proportion of energy coming from fat (22 EN% compared with 33 EN%, respectively = Δ11%) and carbohydrates (53 EN% compared with 47 EN% = Δ6%, respectively) served. The average cost per day for ingredients to prepare the slow UPF ad libitum diet was estimated to be €37,- compared with €36,- for the fast UPF ad libitum diet. These costs were calculated based on the ad libitum amounts and costs of ingredients purchased at a large Dutch supermarket chain. On the basis of the Dutch Healthy Diet Index, the average diet quality (based on 12 items, 120 max score) of both diets was 70 ± 11 and the average diet quality of participants at baseline [food frequency questionnaire (FFQ) based] was 69 ± 17 [28]. Details on Nova classification and energy content of the diets at the level of the meal can be found in Supplementary Table 1. A detailed description of the foods, menu’s and composition of the 2 diets is provided elsewhere [20].

### Experimental procedures

Participants entered the study for a total of 7 wks (Figure 1). The first week included baseline measures of dietary intake (3-day food diary and FFQ) [30], exercise (accelerometry) and body composition [dual-energy X-ray absorptiometry (DEXA)]. The following 2 weeks consisted of the first of 2 dietary intervention periods (UPF Fast-ER, UPF Slow-ER). Within this 2-week diet period, all food (i.e., 3 main meals and all snacks) was offered ad libitum, equivalent to 3 times a standard Dutch meal portion [31]. Meals were served with a glass of water (240 mL), and at breakfast an additional cup of coffee or tea was served (130 mL) [32]. Participants were instructed to eat in their normal way and eat as much or as little as they liked until they felt comfortably full. On weekdays, participants consumed all of their main meals in individual cubicles in a common dining room at the eating behavior laboratory in the Human Nutrition Research Unit at Wageningen University. Participants consumed their breakfast between 08:00 and 08:45, their lunch between 12:30 and 13:15, and their dinner between 17:00 and 17:45. Throughout the day, participants were allowed to drink water from a water bottle (provided by the researchers) or coffee or tea (without sugar or milk) and were asked to record their fluid intake away from the laboratory. Participants were provided with a snack package that was aligned with the arm of the study they were completing (UPF Slow-ER/UPF Fast-ER). They received the snacks after each main meal and were asked to note the time they consumed snacks and return the (empty) packaging and leftovers to record snack intake.

On weekend days, participants were instructed to continue their meal protocols in their home. During the run-in week, a researcher visited the participants in their home to give instructions about meal-setting, meal storage and to provide standardized plates, bowls, glasses, and cutlery. Each Friday, participants received a package with fully prepared and packaged ad libitum meals and snacks, together with beverages (2 cans of soda or 0.0% beer; the same for both diet periods). Participants were provided instructions on how to reheat the meals and asked to record the time of consumption and take pictures of their meals preconsumption and postconsumption. Participants returned all of their weekend meal packages, complete with any leftovers and empty packaging, to determine intake over the course of the weekend.

To confirm that the textural properties of the diets led to a relatively slower and faster ER of the meals, eating behavior was measured during consumption of each meal of each diet arm. On weekdays, participants were video recorded when eating all meals using an integrated webcam camera (ThinkPad) and Action Camera (EKEN H9R, Action cam). Participants could not see themselves being recorded, and post hoc behavioral annotation of eating microstructure was completed to derive the eating behaviors using the behavioral annotation software ELAN (version 6.0 Max Planck Institute for Psycholinguistics, the Language Archive, Nijmegen, The Netherlands). Videos were annotated by trained video coders using a coding scheme developed and validated previously [26]. ER was determined from video by observing start (first bite) and stop (last swallow) of the meal to calculate meal duration. ER was then calculated by dividing the amount consumed by meal duration (min). In line with previous research, ER of meals consumed outside the laboratory (during weekends) was estimated using time stamp data from the online appetite questionnaires that participants completed before and after meals in on their smartphone [16].

To estimate changes in subjective appetite feelings, participants were asked to rate their appetite before and after eating each meal. Before each meal, participants rated their “hunger,” “fullness,” “thirst”, “desire to eat” and "prospective consumption" on a 100 mm line scale anchored from “not at all” to “extremely” in an online questionnaire [33]. To determine meal-to-meal variations in subjective liking and familiarity estimates, participants were asked to consume a single bite at the beginning of each meal, and rate their “liking” and “familiarity” on the same 100-mm line scale. To assess overall satisfaction with the intervention diets, participants completed the diet satisfaction questionnaire at the end of each diet [34].

Body composition measures were conducted before and after each diet. Whole body and regional lean and fat mass (FM) were measured by DEXA scan (Lunar) for each tissue, the ratio of the attenuation 35 keV/61 keV was calculated, and bone, fat and fat-free (FFM) tissue mass were calculated using Prodigy Pro package with 39ncore V18 SP4.1 software. Body weight was measured every Monday, Wednesday, and Friday when on the diet, in the morning after an overnight fast and before breakfast.

Participants were free-living, and physical activity was standardized such that participants were asked to maintain their usual physical activity routine of <4 h/wk (inclusion criteria) and to keep this routine consistent throughout the study. To compare active energy expenditure (kcal/d) between the diets, participants wore an ActigraphTM on their hip and an ActivPAL monitor on their leg during the run-in week, both diet periods, and the washout period to measure fluctuations in sedentary energy expenditure and monitor physical activity.

### Compliance

To retain ecological validity while maintaining control over the dietary manipulation and study outcomes, we chose a semiresidential study design. Participants consumed the majority of their meals in a controlled setting at the Human Nutrition Research Unit in the Division of Human Nutrition and Health, but were free to work and return home in the evenings. This design enabled control of the diets while facilitating normal living patterns, but required additional compliance measures to reduce risk of non-study food and beverage consumption away from the laboratory. Meal intake was measured through pre-meal and post-meal weighing, in line with best practice [35]. Throughout the 2-diet intervention periods, participants wore a continuous glucose monitor to monitor changes in conductivity in the interstitial fluid as a proxy measure of glucose concentration, and as a psychological motivation to adhere closely to the study guidelines. Participant physical activity was monitored throughout the trial using duplicate accelerometry approaches and physical activity-related questionnaires. Participants were asked to report any non-study foods or caloric beverages consumed during the intermeal period (i.e., between the main meals). For the per-protocol analyses, diet days were excluded if non-study food caloric intake exceeded 350 kcal/d. This led to a total of 7 menu days removed from the final analysis. In total, the analysis included 1332 menu days comprising a total of 3444 meals. On weekends, participants were asked to collect 24-h urine samples that were returned to the research team and were the basis for further biochemical compliance measures. Weekend protein and sodium intakes were estimated from measured nitrogen and sodium concentrations in urine, and compared with measured food intakes in the 24-h period prior to the urine collection to ensure alignment [36].

### Study outcomes

The primary outcome of the RCT was average daily energy intake (kcal/d). Secondary outcomes were appetite (changes to reported fullness, hunger, desire to eat, and thirst pre-main and post-main meals) and body composition changes (total bodyweight, FM, and FFM, in kg). Other secondary and exploratory outcomes of the RCT not described in the current manuscript included changes to glucose homeostasis, satiety responsiveness, inflammatory and leaky gut markers, and changes in microbiome composition and functionality. More detail on the rationale, hypotheses, measures, their timing, and outcomes can be found elsewhere [20].

### Sample size

A power analysis was conducted in G∗Power (Windows version 3.1.9.7), indicating that 39 participants were required to achieve a 1-β power of 80% to detect significant differences at *α* = 0.05, 2 tailed. The analysis was based on an estimated effect size of 130 kcal/d with an SD of the difference of 280 kcal/d, giving a Cohen’s *d* of 0.46, which represents a moderate effect size.

### Randomization and concealment of study aim

Participants were randomly assigned in time blocks, and each block lasted 7 wks from the run-in week to study completion. Each block consisted of 2 groups (max group size: *n =* 4), with 1 on each diet order (slow UPF diet > fast UPF diets or fast UPF diet > slow UPF diets). Eligible participants were randomly allocated to a diet order (1:1) by means of randomization envelopes stratified by block. Participant enrolment and allocation sequence were completed by 2 researchers who were both responsible for study oversight.

Each diet consisted of 7 unique daily menus that were presented twice within each diet period. To minimize potential bias due to a menu effect, the order of the day menus was semirandomized (separately for weekday and weekend menus) and then randomly allocated to the blocks. All participants received all-day menus, but in a different order and a maximum of 4 participants received the same menu sequence.

Research has shown that awareness of study goals and food intake measures could potentially influence participant behaviors [37]. To reduce risk of an awareness bias, participants were provided a cover story during recruitment to conceal the true aim of the study. Before the study, participants were informed that the study aimed to investigate the effects of different protein types on digestibility, body composition, and endocrine responses, and that the study was not a weight loss study. After the trial participants were asked to report what they thought was the true aim of the study. In total, 4 of the 41 participants included in the data analysis guessed that the true study aim of the study was related to a texture manipulation or eating speed of processed foods. Participants were informed about the true aim of the study after all participants had completed the trial.

### Statistical analysis

Statistical analyses were completed using SAS (SAS version 9.4; SAS Institute) and statistical significance was set at the 95% confidence interval (CI; *P* values < 0.05). A detailed statistical analysis plan including data analysis script was preregistered in OSF (https://osf.io/afmkh). Outcomes reported in text and tables are reported as mean (95% CI) unless otherwise stated. Before data analyses, normality of the data was visually inspected (histogram) but none of the outcomes reported were non-normally distributed. Analyses to determine the outcomes of the study were conducted on a per-protocol basis, as preregistered; therefore, the single participant who was lost to follow-up (*n =* 1) was excluded as well as individual data on the days (*n =* 4, d) on which participants reported to have consumed ≥ 350 kcal/d outside of the study diets and the days on which a participant switched weekend-day menus (*n =* 2). Hours of sleep per night was not correctly recorded on 5 nights, and these were excluded in the analyses. Per-protocol data were selected during a review of the blinded data and prior to data analyses. Unblinding of the data was only done after analysis of the primary outcomes was completed in duplicate by 2 researchers (ML, MvB).

A repeated measures mixed model was used to test for the main effect of diet (PROC MIXED). The model included diet (UPF Fast-ER, UPF Slow-ER) and diet-day (1–14) and their interaction as fixed factors and random variables included participant, block, diet order, and day menu. The AR1 covariate structure was selected based on the likelihood ratio test. Chi-square comparisons showed that AR1 was the better fit compared with UN, CS, and VC covariate structures (CS compared with AR1, χ^2^ = 61.8, *P* < 0.001). If main or interaction effects were significant, a Tukey corrected t-test was done to compare diets and diet days. For the diet∗diet-day interaction, a contrast was applied to only compare the same days of both diets (i.e., day 1 Slow-ER compared to day 1 Fast-ER diet). The secondary outcomes were analyzed using the same model; however, a few adaptations were made due to time points at which the data were collected and by adding covariates (liking and fat content of the meals served). As preregistered bodyweight, FFM and FM outcomes were corrected for total body water content as determined by bioelectrical impedance analysis (Fresenius Medical Care). Exploratory analysis included generalized linear models (PROC GLM) to test whether changes in body weight and FM aligned with differences in daily energy intake corrected for the variables' baseline body weight and baseline energy intake. The same model was used to test the effects of ER (continuous variable), and %fat served per day on daily energy intake. Pearson correlations were done to assess associations between differences in daily energy intake and changes in body weight.

## Results

### Participant characteristics

**FIGURE 2:**
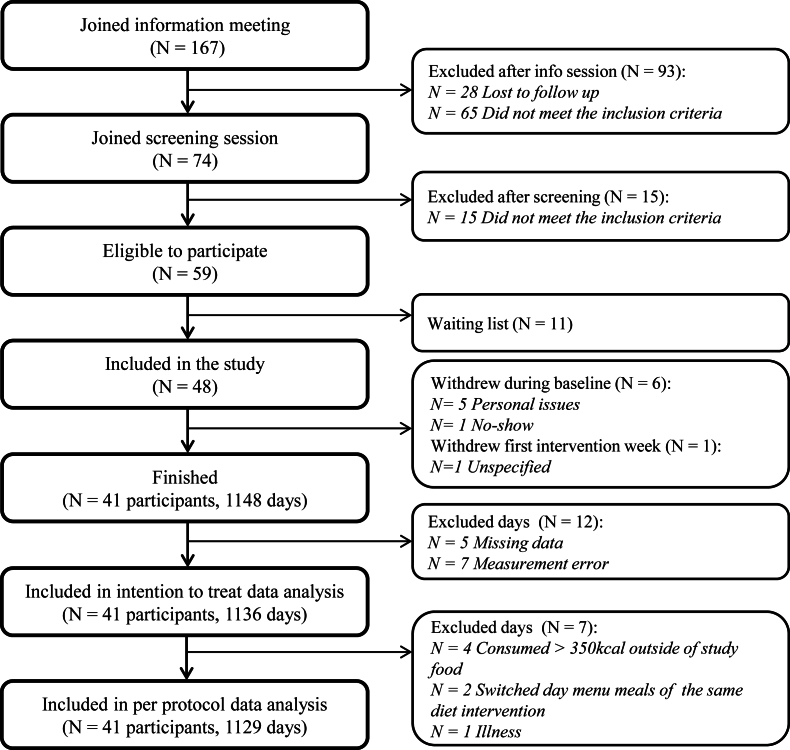
CONSORT study flow diagram.

In total, 167 participants joined the information meeting, of which 59 were eligible to participate in the study of which 11 remained on the waiting list. A total of 48 participants were included in the study; 6 dropped out during the run-in week, and 1 during the first intervention week (Figure 2). Forty-one participants (*n* = 21 male) completed the study, mean (± SD) age 27 ± 5; weight 70 ± 10 kg; BMI 23.4 ± 1.9. All participant characteristics are described in Table 2. On the basis of a 3-day food diary (2 weekdays, 1 weekend d), estimated energy intake during baseline was 2044 kcal/d and 1770 kcal/d during the washout period (Supplementary Table 2).

**TABLE 2:** Participant characteristics (*n* = 41), values are mean ± SD unless otherwise stated

Characteristics	All participants	Male (*n =* 21)	Female (*n =* 20)
Age at baseline (y)	27 ± 5	27 ± 6	27 ± 5
Ethnicity [*n* (%)]			
Non-Hispanic	34 (83%)	17 (81%)	17 (85%)
Hispanic	6 (15%)	3 (14%)	3 (15%)
Unknown	1 (2%)	1 (5%)	—
Race [n (%)]			
Asian	5 (12%)	2 (10%)	3 (15%)
American Indian	2 (5%)	—	2 (10%)
Black or African American	1 (2%)	1 (5%)	—
White	30 (73%)	15 (71%)	15 (75%)
More than 1 race	2 (5%)	2 (10%)	—
Unknown	1 (2%)	1 (5%)	—
Height (m)	173 ± 10	179 ± 8	166 ± 5
Baseline body weight (kg)	70 ± 10	75 ± 9	64 ± 6
FFM (kg)	49 ± 2	57 ± 8	40 ± 4
FM (kg)	18 ± 1	15 ± 4	21 ± 5
Baseline BMI, kg/m^2^	23.4 ± 1.9	23.6 ± 2.0	23.1 ± 1.8
Normal weight^1^ [*n* (%)]	32 (78%)	16 (76%)	16 (80%)
Overweight^2^ [*n (*%)]	9 (22%)	5 (24%)	4 (20%)
Fasting glucose (mmol/L)	4.8 ± 0.4	4.9 ± 0.4	4.7 ± 0.4
HbA1c (mmol/mol)	32 ± 3	32 ± 2	32 ± 3
Blood pressure (mmHg) systolic	116 ± 11	123 ± 9	110 ± 9
Blood pressure (mmHg) diastolic	68 ± 6	68 ± 5	69 ± 7
DEBQ restrained eater score	2.0 ± 0.5	1.9 ± 0.5	2.1 ± 0.6

Abbreviations*:*DEBQ, Dutch Eating Behaviour Questionnaire; FM, fat mass; FFM, fat-free mass; HbA1C, Hemoglobin A1c

1 Includes participants within the BMI range of 21–25 kg/m^2^.

2 Includes participants within the BMI range of 25–30 kg/m^2^.

### Energy intake and ER

**FIGURE 3:**
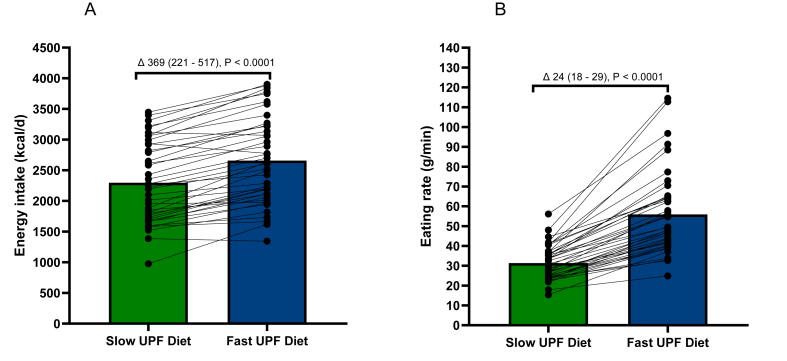
Average daily energy intake (kcal/d) and eating rate of the UPF Slow-ER diet and the Fast-ER diets. Data are presented as means and connected dots are means derived from the same participant, *P* values are derived from linear mixed models with post hoc Tukey corrected 2-sided t-tests, with significance set at *P* ≤ 0.05. (A) Average daily energy intake (kcal/d). Diet had a significant main effect on daily energy intake as an independent variable – diet: F (1, 1051) = 23.98, *P* < 0.0001. (B) Average eating rate of the diets (g/min). Diet had a significant main effect on eating rate as an independent variable – diet: F (1, 835) = 77.27, *P* < 0.0001. ER, eating rate; UPF, ultraprocessed food.

**FIGURE 4:**
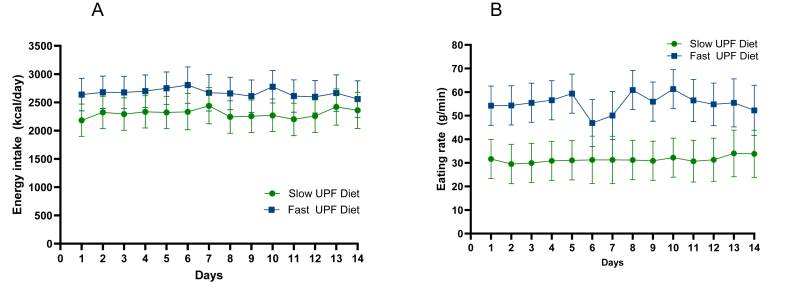
Ad libitum energy intake and eating rate across diet days. Data are presented as means (95% CI), and *P* values are derived from linear mixed models with post hoc Tukey corrected 2-sided t-tests, with significance set at *P* ≤ 0.05. (A) Eating rate (g/min) across the 2-week diet interventions. The diet–time main and interaction effects with eating rate as independent variable were as follows: diet: F (1, 835) = 77.27, *P* < 0.0001; time: F (13, 835) = 1.98, *P* = 0.020; diet∗time: F (13, 835) = 1.04, *P* = 0.415. (B) Energy intake (kcal) per day across the 2-wk diet interventions. The diet–time main and interaction effects with energy intake as independent variable were as follows – diet: F (1, 1051) = 23.98, *P* < 0.0001; time: F (13, 1051) = 1.5, *P* = 0.112; diet∗time: F (13, 1051) = 0.96, *P* = 0.487. CI, confidence interval; UPF, ultraprocessed food.

Participants had a 369 kcal/d (95% CI: 221, 517) lower energy intake when on the UPF Slow-ER diet compared with the UPF Fast-ER diet [diet main effect; F (1, 1051) = 23.98, *P* < 0.0001] (Table 3). All participants responded to the texture intervention by eating faster on the UPF Fast-ER and slower on the UPF Slow-ER diet. The average difference of 43% in ER between the 2 diets led to a sustained effect on intake, consistent across the majority of participants, with 90% (*n =* 37/41) having between 80 and 773 kcal lower daily energy intake (kcal) on the Slow-ER diet compared with the Fast-ER diet (Figure 3A and B; Supplementary Figure 1)**.** This effect was observed across the breakfast, lunch, and dinner meals (Supplementary Figure 2) and was consistent over time (time main effect): F (13, 1051) = 1.5, *P* = 0.112 and no diet–time interaction was observed: F (13, 1051) = 0.96, *P* = 0.486, Figure 4A. Through the use of different meal food textural properties, there was a consistent change to the ER of breakfast lunch and dinner meals of the 2 diets (diet main effect; F (1835) = 77.27, *P* < 0.0001, Supplementary Figure 3). These differences between diets in ER remained consistent over both 2-wk periods of each diet, with the exception of weekend days (diet days 6, 7, and 14), Figure 4B.

**TABLE 3:** Average daily dietary intake (ad libitum, measured intake) and physical activity during the diet periods and at run-in and washout reported as mean (95% CI)

Characteristics	UPF Slow-ER (2-wk diet)	UPF Fast-ER (2-wk diet)	LMM main effect *P* value^1^
Dietary intake patterns			
Daily energy intake (kcal/d)	2301 (2042, 2562)	2671 (2411, 2930)	<0.0001^2^
Daily energy intake (kcal/d)^3^ corrected for liking covariate [F (1, 1055) = 55.06, *P* <0.001]	2310 (2048, 2572)	2674 (2412, 2936)	<0.0001^2^
Daily energy intake (kcal/d)^3^ corrected for fat served covariate [F (1, 1060) = 11.5, *P* < 0.001]	2331 (2090, 2573)	2641 (2399, 2883)	<0.0001^2^
Daily food intake (g/d)	1481 (1324, 1639)	1746 (1588, 1904)	<0.0001^2^
Water intake with main meals (g)	624 (582, 665)	595 (554, 636)	<0.0001^2^
Daily fluid intake (g)	2452 (2043, 2860)	2320 (1911, 2728)	0.019
Energy density consumed (En/g/day) excl. water	1.55	1.53	na
Energy density consumed (En/g/day) incl. water	1.1	1.1	na
CHO (g), EN%	310 (279, 340), 54%	320 (289, 350), 48%	0.0012^4^
Monosaccharides and disaccharides	89 (77, 101)	130 (118, 142)	<0.0001^2^
Fat (g), EN%	60 (52, 68), 24%	99 (91, 108), 33%	<0.0001^2^
Saturated fat (g)	15 (13, 18)	39 (37, 42)	<0.0001^2^
Protein (g), EN%	110 (99, 122), 19%	103 (92, 115), 15%	<0.0001^2^
Fiber (g), EN%	36 (33, 39), 3%	39 (36, 42), 3%	<0.0001^2^
Sodium intake (mg/100 kcal)	3.8 (3.4, 4.2)	4.2 (3.8, 4.7)	<0.0001^2^
Average Diet Satisfactory score 28 (0–5 score)^5^	2.7 (2.6, 2.9)	2.7 (2.5, 2.9)	0.550
Physical activity patterns			
Sedentary behavior (h/d)	20 (19, 20)	20 (19, 20)	0.238
Physical activity expenditure (kcal/d based on accelerometry)	196 (160, 232)	210 (174, 246)	0.217
Sleep (h.min)	7.29 (7.10, 7.49)	7.35 (7.16, 7.55)	0.133

*Abbreviations:* CI, confidence interval; EN, Energy; ER, eating rate; LMM, linear mixed model; UPF, ultraprocessed food.

1 *P* value of the fixed main effect of the LMM.

2 Main effect *P*-value.

3 *P*-value of the covariate effect on the model.

4 Indicates Tukey-correct *P* value <0.01, post hoc test significance between the Slow UPF and Fast UPF diet.

5 Excluding questions on diet costs and eating out (not applicable to the diet interventions).

Participants consumed 264 less grams of food on the Slow-ER diet compared with the Fast-ER diet (diet main effect; F (1, 1051) = 52.03, *P* < 0.0001). The diets did not differ in nonbeverage energy density based on meals consumed, nor when taking account of the water consumed within each meal (meal ED). Average daily water intake differed by 132 g between the 2 diets (F (13, 1060) = 5.46, *P* = 0.019) and this did not lead to differences in dietary energy density as consumed. The macronutrient contributions to the differences in caloric intake are reflective of the diets as served, with a 9% difference in fat EN% consumed between the 2 diets (F (13, 1061) = 1288, *P* < 0.0001). Exploratory analysis confirmed that energy intake was driven by ER (*P* < 0.0001) rather than fat EN% (*P* = 0.082). Correcting for the total amount of fat served did not change the difference in kcal consumed between the 2 diets (2331 compared to 2641 kcal for the slow and fast diet, respectively) (Table 3).

### Hedonic qualities of the diets and subjective appetite ratings

**FIGURE 5:**
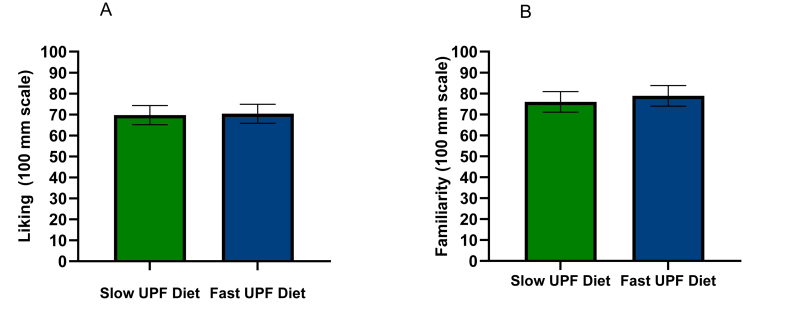
(A) Liking and (B) familiarity ratings of the 2 diets (line scale, 0–100 mm). Liking–diet: F (1, 1051) = 0.28, *P* = 0.596. Familiarity –diet: F (1, 1051) = 1.88, *P* = 0.17. UPF, ultraprocessed food.

The diets were equally liked and rated within the upper part of the liking scale with an average of 70 mm (65–75 mm) on a 100 mm line scale, diet main effect: F (1, 1051) = 0.28, *P* = 0.596. Diets were also equally familiar to the participants [diet main effect: F (1, 1051) = 1.88, *P* = 0.170; Figure 5]. On the level of the meal, UPF Fast-ER breakfast meals were rated as slightly more palatable [7 mm (5−9 mm) on a 100 mm scale] compared with the UPF Slow-ER breakfast meals [F (1, 1003) = 54.94, *P* < 0.0001]. Conversely, the UPF Slow-ER dinner meals received slightly higher liking scores [5 mm (3–7 mm)] compared with the UPF Fast-ER dinner meals [F (1, 1087) = 18.6, *P* < 0.0001]. Whereas meal liking did explain variance in energy intake [liking covariate effect: F (1, 1055) = 55.06, *P* < 0.0001], this effect was similar across both diets. After correcting for liking, the difference in energy intake on the UPF Slow-ER diet was still −365 (95% CI: 412, 315) kcal/d lower compared with the UPF Fast-ER diet. Diet satisfaction scores were the same for both diets [F (1, 40) = 0.36, *P* = 0.550] (Table 3) [34]. Appetite ratings pre-breakfast and post-breakfast, lunch and dinner meals were similar. A few of the ratings were significantly different between the 2 diets but all differences were within the 1–5 mm range on a 100 mm scale and therefore considered negligible [38] (Supplementary Table 3).

### Body weight and composition

**FIGURE 6:**
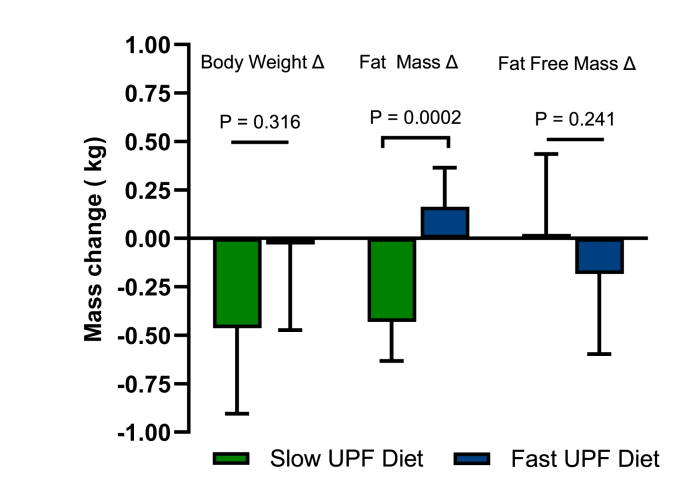
Average body weight and body composition changes (kg) pre-diets to postdiets. Body weight–diet∗pre/post: F (1, 119) = 1.01, *P* = 0.316. Fat mass–diet∗pre/post: F (1, 119) = 14.68, *P* = 0.0002. Fat-free mass–diet∗pre/post: F (1, 119) = 0.272, *P* = 0.102. UPF, ultraprocessed food.

There was no significant change in body weight pre-diets to post-diets, F (1, 119) = 1.01, *P* = 0.316, and body weight remained stable over time with no significant differences between the 2 diets [diet∗time interaction: F (7, 592) = 0.64, *P* = 0.720)]. Body FM corrected for water retention (covariate: F(1, 119) = 0.02, *P* = 0.883) decreased on the slow UPF diet by 0.43 kg (0.23–0.63), diet∗pre–post: F (1, 119) = 14.68, *P* = 0.0002. Body FFM did not significantly change pre-diets to post-diets [diet∗pre–post: F (1, 119) = 2.72, *P* = 0.102]. See Figure 6. Exploratory analysis confirmed that changes in body weight [F (3, 37) = 7.8, *P* = 0.0004] and FM [F (3, 37) = 3.6, *P* = 0.023] aligned with changes in daily energy intakes. Changes in body weight were moderately correlated with differences in energy intake between the diets (*R* = 0.58, *P* < 0.0001) (Supplementary Figure 4).

### Physical activity and compliance

Physical activity level, sedentary behavior, and number of sleeping hours were similar across both diet periods (Table 3). Urinary creatine levels were analyzed as a marker for urine completeness and showed that on 11 occasions samples were incomplete (6.7% of the samples) across 6 participants (based on cut-offs: >10 mg/d/kg bodyweight for females and >15 mg/d/kg bodyweight males) [39]. On the basis of the complete samples, the estimated sodium intake was 3.28 (95% CI: 2.71, 3.85) g/d which differed by 13.4% (group-level bias) from our estimated intake [95% CI: 3.72 (3.2, 4.3) g/d] based on the measured intake and nutrition label information. Protein intake estimation based on the complete samples was 102 (91, 114) g/d, and estimated intake was 108 (92, 125) g/d based on the measured intake and nutrition label information (5.9% group-level bias). Both sodium (*r*^2^ = 0.493, <0.0001) and protein urinary biomarkers (*r*^2^ = 0.569, <0.0001) correlated with the measured intake.

## Discussion

This study provides evidence that texture-derived differences in meal ER significantly influence energy intake from diets comprising UPFs with similar nutritional composition and energy density. In line with our hypothesis, we showed that a UPF diet with textures that promote a slower ER led to a substantial mean reduction in dietary energy intake of 369 kcal per day compared with a UPF diet with a faster ER. The effect of food texture on ER and the associated effect on energy intake were consistent across participants and sustained for the duration of the 2-wk diet intervention period. Meals within both diets were equally familiar and liked by participants, and despite consuming significantly fewer calories on the slower UPF diet, participants did not report notable differences in appetite sensations before or after meals. We show that food texture properties can have a significant impact on ER and energy intake without compromising meal palatability, and that this effect remains consistent over a 2-wk period.

The effect of meal ER on energy intake has been reported previously across numerous acute studies at the level of the meal and the day, and across a wide range of different types of food types, meal occasions, and consumer populations [[40], [41], [42]]. Previous research has demonstrated that a 20% change in ER leads to a proportionate 10% change in acute energy intake [43]. The current trial produced an average difference in ER between slow and fast UPF diets of 43%, which resulted in a 14% difference in daily energy intakes. These findings reinforce and extend previous acute observations, highlighting that texture-based differences in ER can have a sustained impact on energy intake over time, without an observed adaptation in behavior or significant energy compensation. Importantly, this behavioral change occurred when participants were free to consume the meals and diets in their normal way, and without any explicit instruction to eat at a faster or slower rate. In this study, the eating behavior of all participants could be manipulated consistently, without conscious effort, instructions, or a reduction in meal palatability and satisfaction. Food texture cues can influence ER at every meal, and our findings highlight the potential of meal texture-based approaches to be applied to modify ER and guide energy intake in ways that contribute to public health on a larger scale.

Consumption of diets high in UPFs has been associated with many noncommunicable diseases and energy imbalance, and there have been widespread calls to move beyond dietary association and observational studies, to test these putative links with mechanistic studies [44]. If degree of food processing is to be used as a marker of diet quality and inform public health policy, it is first necessary to establish if there are specific characteristics of highly processed foods that contribute to the etiology of noncommunicable disease and positive energy balance. Our findings offer new insights on potential mechanisms underlying previously reported significantly higher energy intakes from experimental UPF diets when compared with minimally processed diets [16]. In that study, the diets differed in meal ER (g/min) and energy intake rate (kcal/min) due to diet differences in textural properties and nonbeverage energy density (kcal/g) [16]. Subsequent studies on the effect of meal ER on food intake suggested that texture-derived differences in meal ER, rather than degree of food processing, were responsible for observed differences in food and energy intakes [18,19]. The results of the current trial support this, and show that texture-derived differences in ER alone accounted for a 369 kcal change in energy intake from diets composed almost entirely of UPF meals [∼95% of dietary energy (kcal)]. This suggests that meal ER can have a sustained impact on energy intake, and may have been a confounder in the previous Hall RCT comparison between UPF and minimally processed diets [16,17,45]. Previously reported differences in energy intake from UPFs could plausibly be attributed to the combined effect of ER and energy density, rather than any specifically defined industrial processes or formulation. With increasing evidence that meal texture and ER are major drivers of energy intake, strategies to manipulate meal textures through guidance on choice, meal alternatives/substitutions or product reformulation could be embraced by mainstream public health [46].

Whereas softly textured, rapidly consumed foods with high energy density can promote higher energy intakes, less is known about the potential to support reductions in acute energy intake through combinations of lower energy density and slower ERs. A recent acute study showed that combinations of ER and energy density (energy intake rate, kcal/min) can both increase and decrease energy intake for meals of equal palatability. Through the manipulation of the energy intake rate (kcals/min), ad libitum lunch meal intake could be increased by 19% (179 kcal) or decreased by 59% (394 kcal) compared with an average lunch meal [27]. Previous research has shown that decreasing energy density has a strong and linear effect on daily energy intakes with minimal adjustments of intake at subsequent meals [47,48]. Taken together with findings from the current trial, this suggests that texture-derived differences in ER could be combined with reductions in energy density to produce a sustained effect on energy intake over time. Sensory-derived eating behaviors can inform how we eat, and therefore complement nutrient reformulation (what we eat), to cumulatively influence intake across meals and over time.

Our trial had several strengths that give confidence in the observed effects and extend the practical implications of our findings. By directly measuring rather than estimating intake, we are confident that reported differences can be attributed to the manipulated variable (ER) between the 2 diets. In addition, our diets are representative of the food environment in the Netherlands and were composed of commercially available foods reflecting existing variations in food textures that can be exchanged or substituted without the need for special preparations, formulations, or equipment. Previous research has further demonstrated that meal ER differs considerably across minimally processed and UPF across a wide variety of everyday food items [17]. This highlights that faster ERs are not unique to UPFs, and that it is possible to substitute similar commodities with different ERs across different degrees of processing [17]. Despite considerable variation in food textural properties in the current trial, food palatability was rated as hedonically appealing and equivalent for both diet arms. Meal “liking” influenced energy intake at the individual participant and meal level, but this effect was the same across both diets. Consumers have a broad acceptance of food texture properties, and these findings suggest that texture-based differences in ER could be implemented within a diet without compromising on liking or diet satisfaction. To be able to isolate the effect of texture and ER on energy intake, the intervention diets were closely matched for many factors known to influence intake. We prioritized matching diets for nonbeverage energy density due to its dominance in driving differences in energy intake [49,50]. This resulted in diets that differed in EN% served and consumed from fat. When the effect of differences in %EN from fat served was evaluated post hoc, the results show that %EN Fat did not explain observed energy intake differences between the 2 diets. This is in line with findings that suggest macronutrient differences served between diets did not offset the impact of ER on energy intake [51].

ER differences tended to be smaller when measured remotely at weekends, and this is likely attributable to differences in measurement methods (online reporting of start and end of the meal compared to direct observations based on video annotations). The semiresidential design of the study allowed participants to be away from the eating behavior laboratory and improve ecological validity while still permitting high control on food intake through multiple compliance measures. Physical activity levels and intake outside of the study foods did not differ between conditions, supporting that the changes were due to the dietary texture-derived ER intervention. Fixed portions of snacks aligned with each study arm were offered to participants to reduce the consumption of non-study foods, and results show no difference in energy consumed from snacks between the diets. Future research is needed to better understand whether the differences observed in our trial would attenuate over a longer period of time and to establish why the intervention led to a larger effect size for some participants compared with others. Results to date suggest an average 2-fold change in ER produces a significant change in energy intake, though this ratio likely differs between individuals and across food categories. Texture-derived differences in ER tend to have a similar effect on individuals that habitually eat a faster or slower rate [[52], [53], [54]], but further research is needed to clarify whether this holds over the longer term, and across different populations of consumers. Understanding which food sensory attributes drive ER and impact energy intakes over the longer term will be key to help tailor successful reformulation strategies or dietary and eating behavior guidelines.

We chose to focus on texture variations within the existing Nova 4 (UPF) category and not to include a minimally processed diet arm for comparison in the current trial, as we believe a diet composed of minimally processed foods could be misconstrued as a “control” and may lead to unrealistic comparisons. Consuming a diet comprising >90% of foods that would be classified as minimally processed by the Nova scheme does not reflect a commonly consumed diet in the Netherlands, and could constitute a stronger intervention that could lead to biased comparisons. Moreover, in prioritizing the matching for palatability and nonbeverage energy density, this becomes impractical as matching minimally processed and ultraprocessed diets on all factors that may influence energy intake is challenging and would likely lead to unrealistic or disliked meals. Epidemiological comparisons highlight with increasing frequency the role of specific food groups within the UPF category in driving association between higher UPF consumption and health effects [[55], [56], [57], [58], [59]]. Our findings are important because they contribute to the growing body of literature that shows the effect of UPF on food intake and highlight that this may vary depending on the textural properties of the foods served. Findings indicate the limitations of treating UPF as a homogenous classification and also help provide guidance on which texture properties are most important in moderating food intake. Our results reiterate concerns that Nova 4 (UPF) is not a coherent categorization due to the wide diversity of nutrient and sensory differences encapsulated within such a broad classification, and the associated diversity in potential health-related effects [60,61].

Average dietary energy intakes on both diets were within the reported and recommended range for adults in the Netherlands. Despite sustained differences in daily energy intakes over the 14 d of intervention between the 2 diets, these differences did not result in excess energy intake or significant changes to body weight, though we did observe a small difference in changes to body composition between the diets. This may be due to the close matching of energy density between the 2 diets compared with previous studies, and the short duration of the diet intervention (14 d). Differences in energy intake between the diets aligned with differences in body weight and FM. Future research is needed to better elucidate the potential of this approach to sustain energy intake reductions that could support changes in body weight. Establishing approaches that effect a longer-term sustained change in food choice and intake behavior remains one of the biggest challenges in nutrition and public health today [62,63]. A limited body of previous research has demonstrated that training adolescents with obesity to eat more slowly using a computerized device results in both a clinically significant reduction in BMI and renormalized endocrine responses [64,65]. Future studies are needed to explore whether sustained adherence to a texture-modified diet that slows ER can have the same effect and support weight loss and improved weight loss maintenance over the long term for better weight management.

In conclusion, we show that food texture-derived reductions in meal ER can significantly reduce daily energy intakes for diets dominated by UPFs. We highlight the sustained effect of food texture in moderating ER and energy intake and suggest further opportunities to apply differences in food texture to support healthy dietary habits.

## Author contributions

The authors’ responsibilities were as follows – CGF, MPL: designed the research; LAJH, ZL, MvB: conducted research; MPL: analyzed data; CGF, MPL: wrote the article; and all authors: read and approved the final manuscript.

## Data availability

Data described in the manuscript, code book, and analytic code will be made publicly and freely available without restriction at OSF (https://osf.io/afmkh).

## Funding

This research is supported by the Dutch Top-Consortium for Knowledge and Innovation Agri & Food (TKI-Agri-food) Project Restructure (TKI 22.150). The “Restructure” project is a public–private partnership on precompetitive research on the influence of food texture and eating rate on energy intake. For more information, go to https://restructureproject.org/.

## Conflict of interest

CGF is a member of the Global Independent Nutrition Advisory Board of Lesaffre; reports a relationship with Kerry Taste and Nutrition that includes travel reimbursement; reports travel reimbursements from USDA, International Life Sciences Institute, Ajinomoto Co. Inc, British Nutrition Society, Nestlé Nutrition Institute, AB Mauri, and the Institute for the Advancement of Food and Nutrition Sciences. He also reports speaking and lecture fees, as well as travel reimbursements, from the World Sugar Research Organization and Northern Irish Dairy Council, and speaking and lecture fees from Ferrero, PepsiCo Inc, General Mills Inc, and Mondelez International Inc. MPL reports speaking and lecture fees, as well as travel reimbursement, from Nestlé Nutrition. KdG is a member of the Global Independent Nutrition Advisory Board of the Mars company. All other authors report no declaration of interests.
