# Supplementary material for: Eating rate has sustained effects on energy intake from ultraprocessed diets: a 2-week ad libitum dietary randomized controlled crossover trial
Source: Am J Clin Nutr. 2025 Nov 26;123(4):101122. doi: 10.1016/j.ajcnut.2025.11.012 (PMC13084570; doi:10.1016/j.ajcnut.2025.11.012)
Supplement: Multimedia component 1 [file mmc1.docx]

# Eating Rate has Sustained Effects on Energy Intake from Ultra-Processed Diets: A Two-Week *Ad Libitum* Dietary Randomized Cross-over Controlled Trial

*Forde et al.*

# Supplementary Table 1

Table 1. Main meal and snack properties (reported as averages of all meals) of the UPF Slow-ER and UPF Fast-ER diets.

|  | UPF Slow-ER | | UPF Fast-ER | | Average of both diets | |  |
| --- | --- | --- | --- | --- | --- | --- | --- |
| **Breakfast** | | | | | | | |
| Portion size (g) | | 1200 | | 1200 | | 1200 | |
| Energy served (kcal) | | 1275 | | 1269 | | 1272 | |
| Energy density (kcal/g) | | 1.06 | | 1.06 | | 1.06 | |
| Ultra-processed foods (EN%) | | 100 | | 82 | | 91 | |
| **Lunch** | |  | |  | |  | |
| Portion size (g) | | 731 | | 732 | | 731 | |
| Energy served (kcal) | | 1712 | | 1716 | | 1714 | |
| Energy density (kcal/g) | | 2.35 | | 2.35 | | 2.35 | |
| Ultra-processed foods (EN%) | | 98 | | 99 | | 98 | |
| **Dinner** | |  | |  | |  | |
| Portion size (g) | | 1393 | | 1394 | | 1393 | |
| Energy served (kcal) | | 2075 | | 2081 | | 2078 | |
| Energy density (kcal/g) | | 1.50 | | 1.50 | | 1.5 | |
| Ultra-processed foods (EN%) | | 96 | | 98 | | 97 | |
| **Snacks** | |  | |  | |  | |
| Portion size (g) | | 176 | | 176 | | 176 | |
| Energy served (kcal) | | 658 | | 658 | | 658 | |
| Energy density (kcal/g) | | 3.74 | | 3.74 | | 3.74 | |
| Ultra-processed foods (EN%) | | 100 | | 100 | | 100 | |

Abbreviations: ER, eating rate; UPF, ultra-processed food

# Supplementary Table 2

Table 2. Average (Mean (95% CI)) Daily Dietary Intake (based on 3-day food diary data) and Physical Activity during the run-in and washout period.

| **Characteristics** | **Run-in week**  **(1 week)** | **Washout period**  **(2 weeks)** |
| --- | --- | --- |
| **Dietary intake patterns** | | |
| Daily energy intake (kcal/day) | 2044 (1832-2257) | 1770 (1556-1984) |
| Daily food intake (g/day) | 2655 (2289-3022) | 2184 (1816-2552) |
| Daily water intake (g) | 2194 (1865-2522) | 1771(1440-2103) |
| CHO (g), EN% | 207 (182-234), 41% | 190 (164-217), 46% |
| Mono- and disaccharides | 75 (64-86) | 67 (56-78) |
| Fat (g), EN% | 90 (79-100), 39% | 74 (63-84), 37% |
| Saturated fat (g) | 29 (26-33) | 26 (22-29) |
| Protein (g), EN% | 86 (75-96), 18% | 71(60-81), 17% |
| Fiber (g), EN% | 23 (19-27), 2.1% | 20 (15-24), 2.1% |
| Sodium intake (mg/100 kcal) | 1.1 (0.9-1.2) | 1.1 (0.9-1.2) |
| Daily alcohol intake (g) | 3.3 (0.9-5.8) | 2.9 (0.4-5.4) |
| Average Diet Satisfactory score 28*  (0-5 score) | 3.1 (2.9 – 3.2) | *NA* |
| **Physical Activity Patterns** | | |
| Resting energy expenditure (kcal/d), (Mean ± SD) | 1628 ± 43 | *NA* |
| Sedentary energy expenditure (hrs/day) | 18 (16-20) | 20 (18-22) |
| Physical activity expenditure (kcal/d based on accelerometery) | 189 (-10 – 288) | 182 (-16 – 381) |

* excluding questions on diet costs and eating out (not applicable to the diet interventions)

Abbreviation: EN, Energy

# Supplementary Table 3

Table 3. Pre and post main meal (breakfast, lunch, dinner) appetite ratings of the UPF Slow-ER and UPF Fast-ER diets.

|  | | | | **ANOVA fixed effect p-value** |
| --- | --- | --- | --- | --- |
| Appetite characteristic (mm, 100 mm line scale) | Pre/ post meal | UPF Slow-ER | UPF Fast-ER | Diet |
| **Breakfast** |  |  |  |  |
| Fullness |  |  |  |  |
|  | Pre | 20 (16-24) | 20 (16-24) | 0.458 |
|  | Post | 74 (71-77) | 76 (73-79) | 0.002 |
| Hunger |  |  |  |  |
|  | Pre | 67 (59-74) | 68 (61-75) | 0.107 |
|  | Post | 15 (13-17) | 14 (12-17) | 0.346 |
| Prospective consumption |  |  |  |  |
|  | Pre | 62 (56-69) | 65 (58-71) | 0.004 |
|  | Post | 19 (16-22) | 18 (14-21) | 0.044 |
| Thirst |  |  |  |  |
|  | Pre | 60 (56-65) | 62 (57-67) | 0.138 |
|  | Post | 25 (20-29) | 20 (16-25) | <0.001 |
| Desire to eat |  |  |  |  |
|  | Pre | 64 (56-72) | 65 (57-74) | 0.154 |
|  | Post | 15 (12-18) | 14 (11-17) | 0.161 |
| Palatability | Pre | 67 (62-72) | 74 (69-79) | <0.001 |
| Familiarity | Pre | 78 (73-82) | 80 (75-84) | 0.058 |
| **Lunch** |  |  |  |  |
| Fullness |  |  |  |  |
|  | Pre | 22 (18-25) | 22 (18-26) | 0.721 |
|  | Post | 74 (71-77) | 75 (72-79) | 0.082 |
| Hunger |  |  |  |  |
|  | Pre | 67 (59-76) | 67 (59-75) | 0.526 |
|  | Post | 16 (14-18) | 14 (12-16) | 0.003 |
| Prospective consumption |  |  |  |  |
|  | Pre | 64 (57-70) | 65 (58-71) | 0.363 |
|  | Post | 20 (17-23) | 18 (16-21) | 0.023 |
| Thirst |  |  |  |  |
|  | Pre | 49 (44-54) | 48 (43-54) | 0.474 |
|  | Post | 30 (25-34) | 27 (23-31) | 0.012 |
| Desire to eat |  |  |  |  |
|  | Pre | 65 (56-75) | 66 (56-75) | 0.604 |
|  | Post | 17 (14-19) | 15 (12-18) | 0.018 |
| Palatability | Pre | 68 (62-74) | 68 (62-74) | 0.917 |
| Familiarity | Pre | 80 (76-84) | 83 (79-87) | 0.010 |
| **Dinner** |  |  |  |  |
| Fullness |  |  |  |  |
|  | Pre | 23 (18-27) | 23 (19-28) | 0.292 |
|  | Post | 80 (76-84) | 79 (75-83) | 0.123 |
| Hunger |  |  |  |  |
|  | Pre | 68 (60-75) | 67 (60-75) | 0.725 |
|  | Post | 10 (9-12) | 11 (9-13) | 0.327 |
| Prospective consumption |  |  |  |  |
|  | Pre | 64 (57-71) | 65 (57-72) | 0.567 |
|  | Post | 15 (10-19) | 15 (11-19) | 0.788 |

| Thirst |  |  |  |  |
| --- | --- | --- | --- | --- |
|  | Pre | 49 (44-55) | 49 (44-55) | 0.919 |
|  | Post | 32 (26-37) | 32 (26-38) | 0.620 |
| Desire to eat |  |  |  |  |
|  | Pre | 66 (56-75) | 65 (56-75) | 0.656 |
|  | Post | 12 (8-16) | 11 (8-15) | 0.189 |
| Palatability | Pre | 74 (70-78) | 70 (66-73) | <0.001 |
| Familiarity | Pre | 80 (76-84) | 83 (79-87) | 0.010 |

Abbreviations: ER, eating rate; UPF, ultra-processed food

# Supplementary Figure 1

Supplementary Figure 1. Difference in average daily energy intake (kcal/day) between the UPF Fast-ER diet and UPF Slow-ER diet plotted for each participant.

# Supplementary Figure 2

Supplementary Figure 2. Average daily energy intake (kcal/day) per main meal and overall snack intake of the UPF Slow ER and the Fast ER-diets. Data are presented as means and error bars show the 95% confident intervals, P-values are derived from linear mixed models with post-hoc Tukey corrected two-sided t-tests, with significance set at P ≤ 0.05. Abbreviations: d, day; UPF, ultra-processed food

# Supplementary Figure 3

Supplementary Figure 3. Average Eating rate (g/min) per main meal and overall snack eating rate of the UPF Slow ER and the Fast ER-diets. Data are presented as means and error bars show the 95% confidence intervals, P-values are derived from linear mixed models with post-hoc Tukey corrected two-sided t-tests, with significance set at P ≤ 0.05.

Abbreviation: UPF, ultra-processed food

# Supplementary Figure 4

Supplementary Figure 4. Plots of the differences in (A) body weight change, (B) fat mass change and (C) fat free mass change (Fast UPF – Slow UPF) and difference in daily energy intakes of the Fast UPF and Slow UPF diets. Data are presented as means and P ≤ 0.05 are considered statistically significant.

Abbreviation: UPF, ultra-processed food
